# Checkpoints are blind to replication restart and recombination intermediates that result in gross chromosomal rearrangements

**DOI:** 10.1038/ncomms7357

**Published:** 2015-02-27

**Authors:** Saed Mohebi, Ken’Ichi Mizuno, Adam Watson, Antony M. Carr, Johanne M. Murray

**Affiliations:** 1 Genome Damage and Stability Centre, School of Life Sciences, University of Sussex, Falmer, Brighton BN1 9RQ, UK

## Abstract

Replication fork inactivation can be overcome by homologous recombination, but this can cause gross chromosomal rearrangements that subsequently missegregate at mitosis, driving further chromosome instability. It is unclear when the chromosome rearrangements are generated and whether individual replication problems or the resulting recombination intermediates delay the cell cycle. Here we have investigated checkpoint activation during HR-dependent replication restart using a site-specific replication fork-arrest system. Analysis during a single cell cycle shows that HR-dependent replication intermediates arise in S phase, shortly after replication arrest, and are resolved into acentric and dicentric chromosomes in G2. Despite this, cells progress into mitosis without delay. Neither the DNA damage nor the intra-S phase checkpoints are activated in the first cell cycle, demonstrating that these checkpoints are blind to replication and recombination intermediates as well as to rearranged chromosomes. The dicentrics form anaphase bridges that subsequently break, inducing checkpoint activation in the second cell cycle.

## Abstract (web summary)

Homologous recombination can overcome replication fork inactivation, but this can cause gross chromosomal rearrangements. Here, the authors show that DNA damage and intra-S phase checkpoints are blind to chromosome rearrangement in the first cell cycle, and are only induced in the second cell cycle.

## Graphical abstract

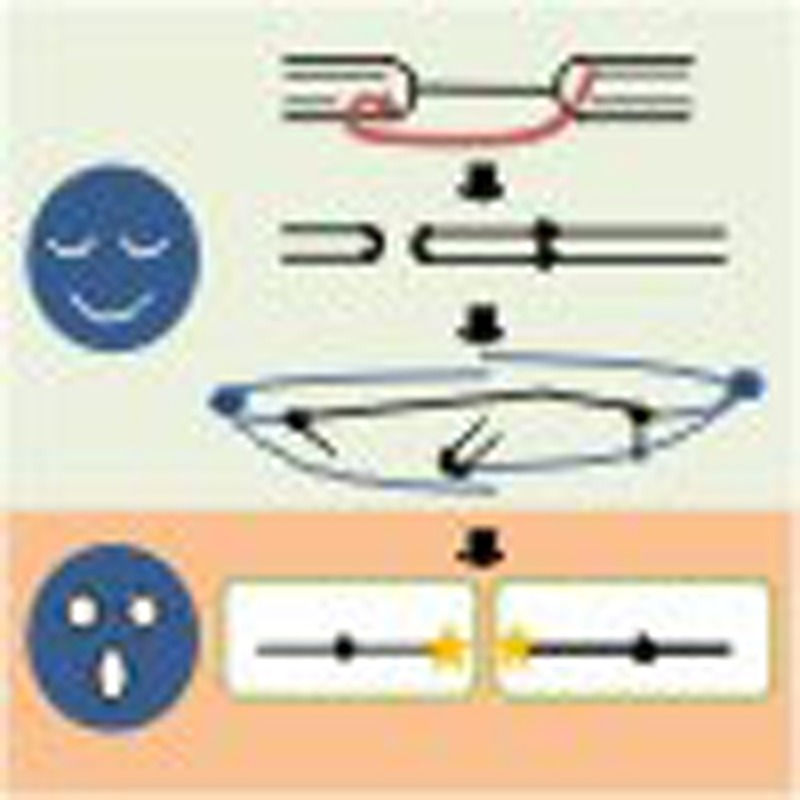

Faithful and complete replication of the genome before chromosome segregation at mitosis is vital for genome stability and cell survival. Some regions of the genome are particularly difficult to replicate due to the presence of replication barriers, including DNA-bound proteins and structured DNA, that lead to replication arrest^1^. Arrested forks can be rescued by the converging fork but, in regions with few origins, inactivated forks must be restarted to avoid underreplication^2^. Replication fork inactivation can be overcome by homologous recombination (HR)^3^,^4^. However, while replication restart using HR is mostly accurate, occasionally the incorrect template is used (non-allelic homologous recombination: NAHR) leading to gross chromosomal rearrangements (GCR)^3^ that contribute to chromosome instability (CIN). Increased replication fork arrest correlates both with genome instability in human microsatellite expansion diseases^5^ and to the chromosome segregation defects (predominantly acentric chromosomes and anaphase bridges) seen in CIN^+^ colorectal cancer cells^6^.

Replication stress is an early driver of oncogenesis and one of the features of oncogenesis is the expression of common fragile sites (CFS)^7^,^8^. CFS were first defined as genomic locations susceptible to breakage in response to mild replication inhibition^9^ and are frequent sites of chromosome rearrangements in cancer. CFS may contain difficult to replicate regions, are often late replicating and contain few origins of replication^10^. CFS instability likely results from a combination of causes including fork arrest, incomplete replication and the processing by structure-specific nucleases of late replication (LRIs) or recombination intermediates arising from replication fork restart^11^. For example, HR-dependent restart involving Rad51-dependent strand invasion and joint molecule formation results in Holliday junctions (HJ). While dissolution of double HJs by the BLM complex (comprising BLM helicase, defective in Bloom's syndrome, with Topoisomerase3 and accessory factor Rmi1) would not result in an exchange of genetic material, resolution of either single or double HJs, depending on the plane of resolution, can generate crossover products resulting in GCR^12^. Both BLM and the Mus81/Eme1 HJ resolvase, which promotes CFS expression, associate with CFS regions after bulk DNA synthesis implicating HR-dependent replication restart as a contributor to GCR in these regions^11^,^13^,^14^.

Chromosome segregation defects are increased in Bloom’s syndrome cells but this has not been correlated with obvious mitotic delay^15^,^16^. While this and several other lines of circumstantial evidence suggest that stochastic replication problems, NAHR-dependent recombination intermediates and the resulting chromosomal rearrangements do not generate robust checkpoint activation leading to mitotic delay, the stochastic nature of replication problems has precluded a definitive analysis of the role of DNA replication and DNA damage checkpoints in delaying mitosis in response to individual replication problems. Previous work in fission yeast using mutants defective in the resolution of recombination shows that cells enter mitosis with a timing similar to wild-type cells following release from replication arrest, but then fail to segregate their chromosomes^17^,^18^. The fact that the segregation defect, but not viability loss, is relieved by concomitant deletion of core HR genes suggests that recombination intermediates *per se* do not generate a checkpoint signal, but this has not been associated directly with HR-dependent replication restart or with NAHR.

In previous work we characterized a site-specific replication arrest system in fission yeast (Fig. 1a). Replication arrests at both sides of a short palindrome and errors during HR-dependent replication restart generate acentric and dicentric chromosomes at a high frequency^19^,^20^. The dicentric chromosomes are sister chromatid fusions that consist of a single DNA molecule and, since the sister chromatids are held together by cohesin, contain a single centromere that bi-orientates (see Fig. 1b). We thus denote these dicentrics as ‘capped’ chromosomes, to differentiate them from dicentric bivalent chromosomes which have two independent centromeres. It should be noted that replication of the capped chromosome would generate a dicentric.

The previously characterized chromosome rearrangements are caused by both NAHR and by the propensity of the restarted fork replicating the correct template to U-turn at the centre of a short inverted repeat present at the locus^19^,^20^. Both mechanisms generate HJ that, depending on the direction of resolution, regenerate either the original chromosome configuration or an acentric and reciprocal capped chromosome (see Supplementary Fig. 1). The centromere of the capped chromosome aligns correctly at mitosis, but forms a bridge at anaphase. The frequency of rearrangements in this system (~30% HJ formation, see below) provides an ideal opportunity to definitively establish if replication arrest and restart by HR, the subsequent recombination intermediates or the rearranged chromosomes themselves can activate the DNA damage or replication checkpoints to delay mitotic entry.

By analysing site-specific replication restart in a single defined cell cycle we show that HR-dependent replication restart occurs in S phase, shortly after replication arrest, and that HR-dependent replication intermediates are resolved into acentric and capped chromosomes in G2. Cells do not delay mitosis and neither the DNA damage nor the intra-S phase checkpoints are activated in the first cell cycle. Thus, these checkpoints are blind to the replication and recombination intermediates as well as to rearranged chromosomes. The capped chromosomes form bridges at anaphase. These subsequently break; inducing checkpoint activation in the second cell cycle, which is dependent on prior passage through mitosis.

## Results

### HR-dependent replication restart does not delay mitosis

**Figure 1:**
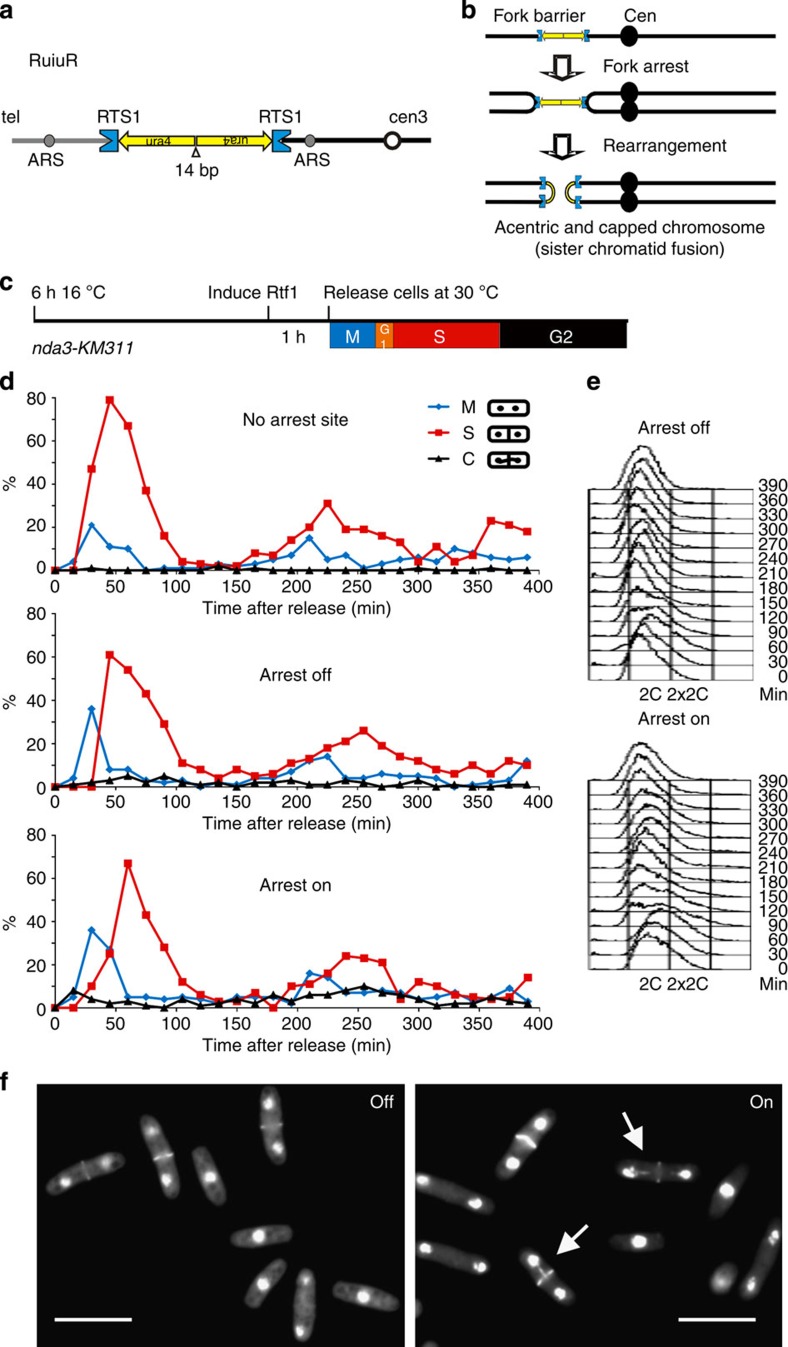
HR-dependent replication restart does not delay mitosis. (**a**) Cartoon of the replication arrest system RuiuR integrated at the *ura4* locus on fission yeast chromosome 3. ARS denotes replication origins, Cen3 centromere 3, tel the telomere. Concave blue boxes represent *RTS1*, replication termination sequences. Yellow arrows represent *ura4* sequences and orientation. Open triangle shows 14-bp interrupting sequence at the palindrome centre. (**b**) HR-dependent restart of replication fork arrest on either side of RuiuR generates reciprocal acentric and capped chromosomes (fused sister chromatids). (**c**) Experimental setup of time course. Log phase cultures were arrested in mitosis by incubation at 16 °C for 6 h, uracil was added to induce Rtf1 60 min before release at 30 °C when sampling started. (**d**) Profiles of cell cycle progression shown by percentage of mitotic (M; binucleate anaphase cells), septated (S) and ‘cut’ (septum bisects the DNA; C) cells in control cultures without the arrest site (top panel), uninduced (− uracil, arrest off) and induced (+ uracil, arrest on) cultures from a representative experiment (*n*>3). After release, all cultures progress through the cell cycle with similar kinetics but ‘cut’ cells, where the septum bisects the DNA, are seen in the mitosis after passage through S phase when Rtf1 is induced (increase of 16.04% over uninduced, *P*=0.0008, Student’s *t*-test). A background level of aberrant cells is seen on release from the mitotic block (0–120 min) this is not statistically different between uniduced and induced cultures (*P*=0.845, Student’s *t*-test). (**e**) Flow cytometry analysis shows both arrest ‘off’ and arrest ‘on’ cultures progress with similar kinetics. In *S. pombe* septation is coincident with S phase so cells move transiently from 2C towards 2 × 2C before the daughter cells separate. (**f**) Examples of cells in the mitosis after arrest showing ‘cut’ cells (arrow) in the induced ‘on’ culture but not in the uninduced ‘off’ culture. Cells were fixed in methanol and stained with DAPI and calcofluor to visualize DNA and the septum, respectively. Scale bar: 10 μm.

In our site-specific replication arrest system, binding of a MYB-related protein, Rtf1, to the *RTS1* sequences induces fork arrest. More than 90% of replication forks arrest at each *RTS1* (ref. 21) and replication of the intervening sequence requires fork restart by HR^19^. We have optimized regulation of Rtf1 transcription^22^ and combined this with a cell cycle synchronization protocol (Fig. 1c) exploiting a cold sensitive beta tubulin mutant (*nda3-KM311*) to examine the timing of rearrangements in two consecutive cell cycles. Induction of stalling in this system leads to an ~16% increase in GCR and the *nda3-KM311* mutant had no effect on induction levels (Supplementary Fig 2). Rtf1 was either induced, or not, 60 min before release from the mitotic block and samples taken at intervals for analysis following release at 30 °C (permissive for *nda3-KM311*). On release, cells rapidly completed mitosis (M) and progressed synchronously through the next cell cycle as judged by mitotic and septation (cytokinesis) indices (Fig. 1d) and by DNA content analysis using flow cytometry (Fig. 1e). Flow cytometry analysis is complicated in *Saccharomyces. pombe*: the increase in propidium staining seen for 60- and 90-min samples characterizes S phase. The initial 2C peak at time 0 represents a single 2*N* mitotically arrested nucleus. After mitosis, the 2*N* peak represents 2 × 1C nuclei in attached daughter cells. The increase from a 2*N* content (60- and 90-min samples) thus represents two replicating (>1*N*) nuclei in a single particle. This >2*N* population rapidly collapses back to the 2*N* peak as the two daughter cells separate into two late S/G2 cells (*T*=120–180). Because G1 is very short in *S. pombe,* septation, which precedes cell separation, is coincident with S phase.

Replication arrest, HR restart and the generation of aberrant chromosomes did not affect cell cycle progression: movement through the first cell cycle displayed equivalent kinetics (M1 to M2 or S1 to S2 ~220 min; Fig. 1d) that were independent of either the presence of the replication arrest locus (top panel) or active arrest (Rtf1 induction; ‘arrest on’ Fig.1d bottom panel). In cultures where replication arrest was active, chromosome bridges and ‘cut’ cells (where the septum bisects the DNA, see Fig. 1f) were significantly increased over the uninduced cultures (‘arrest off’ Fig. 1d middle panel) specifically in the mitosis after replication arrest (16.04% increase, *P*=0.0008, Student’s *t*-test). This was not evident in the mitosis before replication arrest (0.22%, *P*=0.845). Thus, mitotic catastrophe is caused by the capped and acentric chromosomes forming bridges and/or lagging chromosome fragments.

### HR-dependent replication restart generates GCR in G2

**Figure 2:**
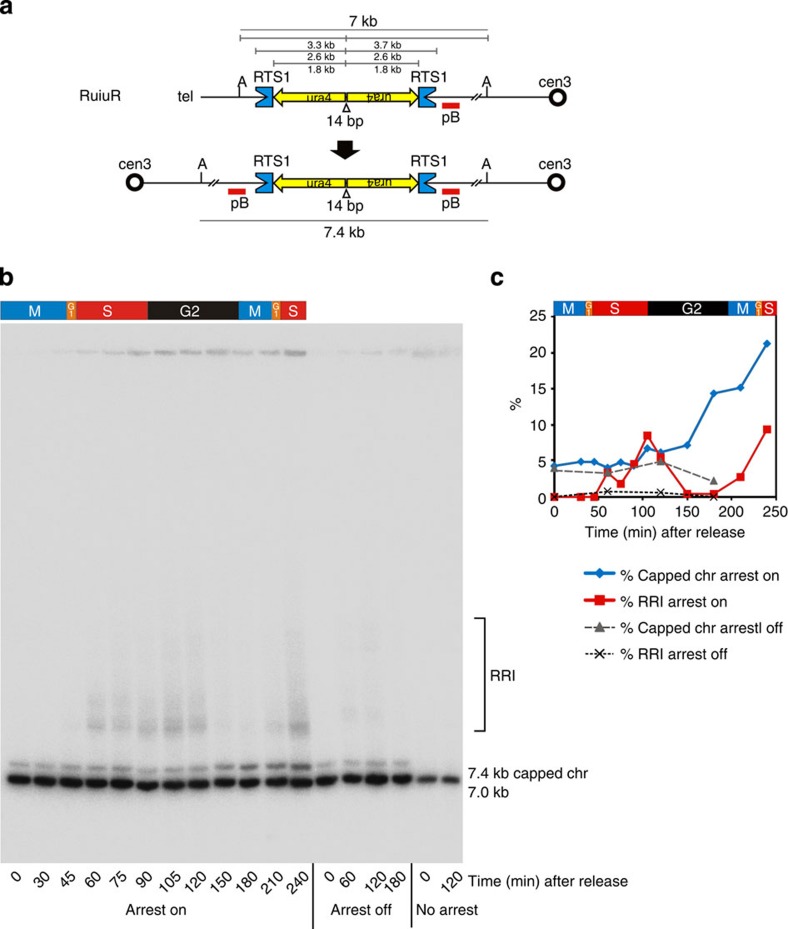
HR-dependent replication restart generates chromosome rearrangements in G2. (**a**) Cartoon of replication arrest system. Cen3 denotes centromere 3. Concave blue boxes represent *RTS1*, replication termination sequences. Yellow arrows represent *ura4* sequences and orientation. Open triangle shows 14-bp interrupting sequence at the centre of the inverted repeat. Red bars represent probe pB. A indicates *Ase*I restriction site. Sizes of initial and predicted capped chromosome *Ase*I fragments are shown. (**b**) Southern blot of samples taken at designated minutes after release from mitotic block with Rtf1 induced (arrest on) or uninduced (arrest off) or no *rtf1* (no arrest; representative experiment, *n*=3). Genomic DNA was digested with *Ase*I and probed with pB. (**c**) Quantification of rearranged fragments in **b**. The 7.4 kb capped chromosome fragment increases from background levels after 150 min. The background level of capped chromosome is also seen in the ‘off’ culture but is absent in the *rtf1* null strain. Slow migrating replication arrest and recombination intermediates (RRI) are seen from 60 to 120 min in the ‘on’ culture, coincident with S phase, reducing as the capped chromosome increases in G2 and returning in the second S phase (210–240 min). RRI are seen faintly in the ‘off’ but are absent in the ‘no arrest’ culture.

To investigate the timing of chromosome rearrangement, samples were taken at 15 min intervals after release from the mitotic block and *Ase*1-digested DNA analysed by Southern blot (Fig. 2a,b). No rearrangements are evident in a ‘no arrest’ *rtf1*Δ control. A background level of a 7.4 kb band, diagnostic of the capped chromosome rearrangement, remains constant for the uninduced ‘off’ culture (Fig. 2b; ‘arrest off’ quantified as ~5%, Fig. 2c). This background occurs because P_urg1_-*rtf1*^+^ expression in uninduced cultures is low, but not zero^22^. Importantly, when replication arrest was induced, the 7.4 kb band began to increase between 150–240 min, reaching ~20%. Comparable kinetics are shown for an independent time course, where DNA was digested with *Bgl*II for better separation of fragments (Supplementary Fig. 3a–c). The levels of induction of GCR are in agreement with the range of levels of rearrangements after a single cell cycle (Supplementary Fig. 2) determined by PFGE of whole chromosomes from independent isolates (15.8±4.8%). Thus ~16% of cells generate a capped chromosome *de novo* in a single cell cycle. In *S. pombe* there is no Gen1/Yen1 (ref. 23) and resolution is mainly dependent on Mus81/Eme1 (ref. 24). While the bias of HJ resolution in the mitotic cycle is not demonstrated, it is proposed that—despite the fact that Mus81/Eme1 resolves nicked meiotic HJs with a bias towards crossovers—the resolution of intact mitotic HJs occurs without orientation bias^12^,^25^. Because rearranged chromosomes only result from the resolution of HJs in one of the two planes (Supplementary Fig. 1), unbiased resolution implies that the ~16% GCR observed reflects a situation whereby ~30% of cells would have contained recombination intermediates.

Branched DNA molecules such as replication or recombination intermediates migrate through agarose gels with decreased electrophoretic mobility compared with linear DNA. The probe used (Fig. 2b,c and Supplementary Fig. 3b,c) hybridized to slower migrating structures that were evident in S phase (60 min after release), peaked at 105 min and declined by 150 min when the capped chromosome band began to increase. The dependence of these slow migrating forms on replication arrest at *RTS1* is consistent with them representing arrest and recombination intermediates that are converted to the linear species (original and capped) in G2.

### HR-dependent replication restart occurs in S phase

**Figure 3:**
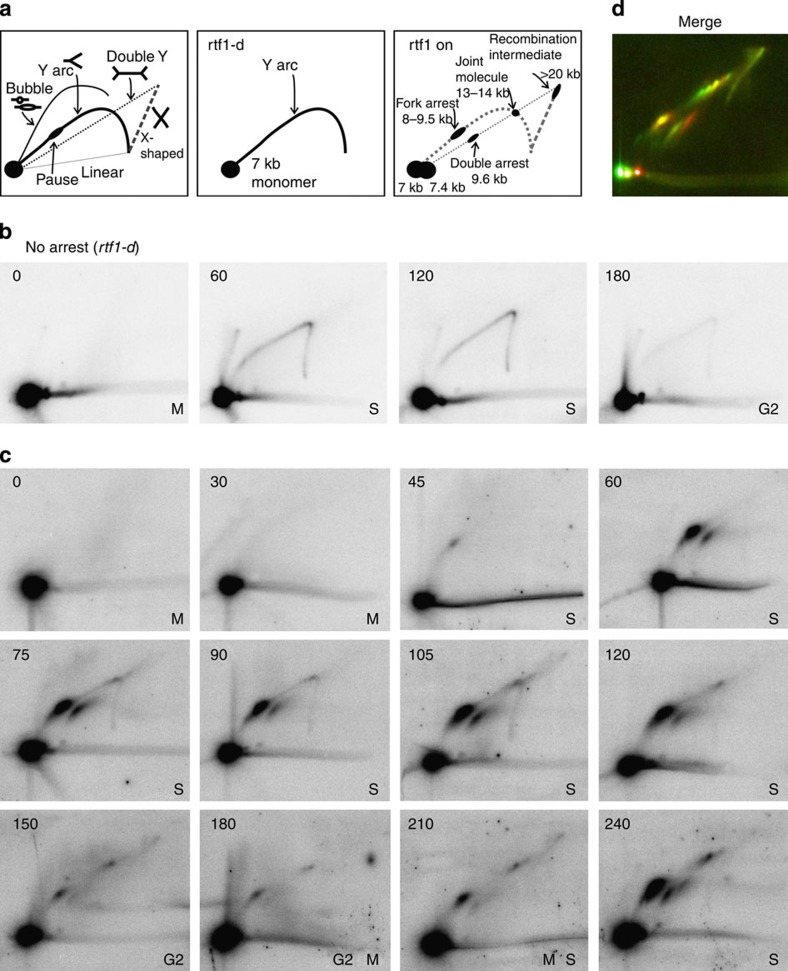
HR-dependent replication restart occurs in S phase. (**a**) Left: cartoon of replication intermediates seen by neutral–neutral 2D gel electrophoresis. ‘rtf1-d’: the Intermediates shown correspond to those visualized with probe pB when the locus is replicated in the absence of replication arrest. ‘rtf1 on’: intermediates corresponding to when replication arrest is induced. (**b** and **c**) Two-dimensional gel electrophoresis of replication intermediates from samples taken at designated minutes after release from mitotic block in the absence of *rtf1* (no arrest; **b**) or with Rtf1 induced (arrest on; **c**; representative experiment, *n*=3). Numbers indicate minutes after release. M/S/G2 indicate cell cycle stage. Genomic DNA was digested with *Ase*I and probed with pB (centromeric to palindrome). In the ‘no arrest’ culture a dominant monomer spot (bottom right), corresponding to unreplicated DNA, and a Y-arc, indicative of passive replication through the region, are seen. The Y-arc is lost when fork arrest induced and a pause spot on the Y-arc indicates replication arrest, a pause spot on the double Y-arc indicates pausing at both *RTS1* sequences. Larger (13–14 kb and >20 kb) HR-dependent joint molecules are seen from 75 min, pause and recombination intermediates are reduced in G2 (150–180 min) but return in the second S phase (240 min), where an additional pause spot is seen to the right of the double pause spot. (**d**) Two-dimensional gel electrophoresis of replication intermediates when fork arrest is induced in an asynchronous culture. DNA was digested with *Ase*I and hybridized sequentially with probes pA telomeric and pB centromeric to the palindrome. In the merged picture the signal corresponding to the telomeric probe is green, the centromeric probe is red (yellow is when both probes hybridize). Red and green spots flanking the yellow monomer spot correspond to capped chromosome and acentric rearrangements. Some recombination-dependent intermediates are detected with a single probe and notably the spot corresponding to the novel pause to the right of the double pause site in the synchronous 240 min sample is only detected by the centromeric probe pB, thus correlating with replication pausing on the capped chromosome in the second cell cycle.

To characterize the replication arrest and recombination intermediates further, DNA was analysed by neutral–neutral two-dimensional gel electrophoresis (2DGE; Fig. 3). In mitotically arrested cultures (*T*=0) only the monomer spot is seen. In *rtf1*Δ cultures (no arrest) in S phase (60 min after release) passive replication is evident from the Y-arc, which was mostly gone by G2 (*T*=180; Fig. 3a,b). In the P_urg1_-*rtf1*^+^ ‘off’ culture, a faint pause site on the Y-arc is evident, consistent with the low basal level of P_urg1_-*rtf1*^+^ (Supplementary Fig. 4). In contrast, when stalling was induced, in early S (*T*=45) small Y molecules accumulated in a pause spot on the Y-arc, consistent with the early centromere–proximal origin initiating replication that subsequently arrested at *RTS1* (Fig. 3a,c). By *T*=60 this arrest site increased in intensity and a second spot running on the double Y-arc was visible. This is consistent with the incoming fork originating from a late firing origin telomere–proximal to the second *RTS1* site, creating a double arrest spot. At 75 min additional spots at the Y-arc apex and on the X-spike appear, corresponding to HR-dependent joint molecules^21^. Quantification of the RI and stalled fork signals relative to the total signal of replication signals (that is, excluding just the monomer spot^21^) showed that, at 105 and 120 min after release, the total pause (single and double) signals represented 84.3% and 82.4% of the total, respectively. This is consistent with previous analysis of asynchronous cultures, where ~90% of forks were arrested at RTS1. Recombination-dependent intermediates made up 8.7% and 5.3% of the total replication signal at 105 and 120 min, respectively. It should be noted that the quantification is an underestimate: unlike in Lambert *et al.*^21^ the samples were not cross-linked, so only stable intermediates were detected. The stall and recombination intermediates remain visible until 120 min and decline from 150 to 210 min (G2-M). Similar structures, plus an additional spot close to the double arrest spot, reappear at 240 min (second S phase). The additional spot was not detected by a probe (pA) homologous to sequences telomeric to the *RTS1* locus (Fig. 3d and Supplementary Fig. 4). Likely it represents fork arrest on the capped chromosome in the second cell cycle. We note that this analysis does not distinguish between a broken or intact capped chromosome in the second cell cycle. Replication of an intact capped chromosome to form a dicentric chromosome and its subsequent segregation at mitosis would drive further genome rearrangements.

**Figure 4:**
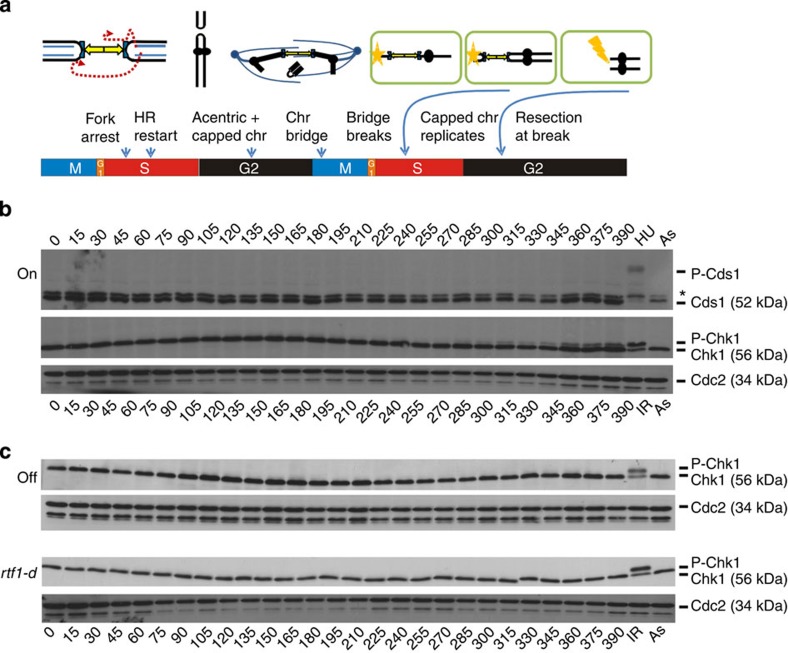
HR-dependent restart and recombination intermediates do not activate the DNA damage checkpoint in the first cell cycle. (**a**) Cartoon of timing of replication arrest and HR-dependent restart generating genomic rearrangements. Replication initiates ~45 min after release and arrested forks accumulate at *RTS1* by 60 min (first arrow). HR-dependent replication restart occurs ~15 min of arrest (within S phase; second arrow) and recombination intermediates accumulate. The intermediates have declined by 150 min (G2; see Fig. 3c) and capped chromosomes start to form (third arrow). Signals are the result of multiple single restart events in individual cells, leading to an overall signal that is dependent on the level of synchrony. Thus, it appears that the resolution of HR intermediates is resisted until G2. The rearranged chromosome is formed before mitosis and gives rise to chromosome bridges (fourth arrow) in anaphase and ‘cut’ cells following septation (cytokinesis; fifth arrow) leading to a one-ended DSB in the daughter cells (sixth arrow). (**b** and **c**) Profile of Cds1^Chk2^ (S phase checkpoint effector kinase) and Chk1 (DNA damage effector kinase) activation at designated times through the cell cycle following release from mitotic block in induced (on; **b**) cultures and uninduced (off; **c**, top panel) or no arrest (rtf1-d; **c**, bottom panel) cultures (representative experiment, *n*=3). HU indicates cells treated with 10 mM HU for 3 h, IR indicates cells treated with 200 Gy and As indicates asynchronous controls. Cds1^Chk2^ was detected using anti-Cds1 antibodies and HA-Chk1 by anti-HA, * indicates a non-specific band. Cdc2 detected by anti-Cdc2 antibodies was used as a loading control. No phosphorylated Cds1^Chk2^ was seen at any time point and no Chk1 phosphorylation was seen in the first cell cycle following release. However, phosphorylated Chk1 was detected from 285 min only when replication arrest was induced, consistent with damage generated in mitosis activating the checkpoint in the second cell cycle.

We have thus determined the timing of events in a single cell cycle (Fig. 4a): replication initiates ~45 min after release, with robust arrest evident at *RTS1* by 60 min. HR-dependent replication restart occurs within S phase (~15 min after arrest) and replication pausing and recombination intermediates are visible for a further 45 min. The overall HR intermediate signal likely represents the accumulation of random restart events in individual cells within the population rather than synchronous restart. The maintenance of recombination intermediates would be consistent with resolution of HR intermediates being resisted until G2 (refs 26, 27). HR intermediates decline after 120 min, coincident with the increase in capped chromosomes in late S and G2. These subsequently give rise to chromosome bridges in mitosis and ‘cut’ cells during septation (cytokinesis), where the septum bisects the DNA. Replication intermediates and replication stall signals return in the second S phase, where a new pause site only detected by centromeric sequences marks the replication of the capped chromosome.

### Checkpoints are not activated in the first cell cycle

Having determined the timing of replication arrest, HR-dependent replication restart and capped chromosome formation in a single cell cycle, we were in the position to establish if these events activate the intra-S phase or DNA damage checkpoints. In fission yeast, the ATR homologue, Rad3^ATR^, is required for both these checkpoints, with Tel1^ATM^ having only a minor role. Within S phase, Rad3^ATR^ and the downstream effector kinase Cds1^Chk2^ are required to stabilize stalled forks and to delay mitotic entry (*S. pombe* Cds1^Chk2^ is the human Chk2 and *Saccharomyces cerevisiae* Rad53 homologue: note in metazoans Chk1—not Chk2—acts downstream of ATR in S phase checkpoints). Rad3^ATR^ and the downstream effector kinase Chk1 respond to DNA damage in G2 to delay mitotic entry. Rad3^ATR^, Cds1^Chk2^ and Chk1 are not required for HR-dependent replication restart and the generation of chromosomal rearrangements^28^ (also see Supplementary Fig. 5a,b). Rad3^ATR^, but not Cds1^Chk2^ or Chk1, is required to limit Exo1-dependent resection behind arrested forks^28^ and the *rad3*Δ mutant showed a slight increase in sensitivity on replication arrest in the palindrome, while no additional loss of viability was seen for *cds1*Δ or *chk1*Δ backgrounds (Supplementary Fig. 5c).

To establish if replication pausing at the two *RTS1* sequences led to activation of the intra-S phase checkpoint, we examined Cds1^Chk2^ phosphorylation, a surrogate marker of activation^29^. Despite >80% of replication forks being stalled at *RTS1*, no pCds1^Chk2^ was evident in either the first or the second S phase after Rtf1 induction (Fig. 4b). The Cds1^Chk2^ phosphorylation assay was capable of detecting pCds1^Chk2^ at a level where 1% of a population of cells are exposed to hydroxyurea; Supplementary Fig. 5d). While checkpoint activation by stalling the majority of replication forks in 1% of cells (hydroxyurea arrests replication through nucleotide depletion) is not directly comparable to the situation where two arrested forks are specifically arrested in >80% of cells, the assay’s sensitivity allows us to conclude that two arrested forks in a single cell are not sufficient to globally activate the intra-S phase checkpoint.

We next analysed the activation of the DNA damage checkpoint by monitoring Chk1 phosphorylation (Fig. 4c). No pChk1 was visible in the first cell cycle following Rtf1 induction. Thus, neither replication fork arrest at *RTS1*, HR-dependent replication restart, the recombination intermediates formed nor the acentric and capped chromosome subsequently arising in G2 can activate the DNA damage checkpoint in the first cell cycle. However, a slow migrating band was visible in the second G2 (from 285 min). Activation of the DNA damage checkpoint in the second cell cycle suggests that DNA damage caused by passage through mitosis generates a checkpoint signal. Consistent with this, inhibition of chromosome segregation in mitosis by the addition of thiabendazole, a spindle inhibitor, reduced Chk1 phosphorylation in the second cell cycle (Supplementary Fig. 6).

We propose that, after breakage of the capped chromosome bridge during mitosis, the daughter cells receive a chromosome fragment with an unrepairable one-ended double-strand break (DSB). Since ~16% of mitotic cells contain anaphase bridges, a similar proportion of daughter cells would contain a single DSB and this is sufficient to generate a detectable DNA damage checkpoint signal. While the replication/recombination intermediates are more numerous than this in the previous cell cycle, they do not result in DNA damage checkpoint activation.

## Discussion

We have used a site-specific replication fork-arrest system in fission yeast to demonstrate that neither recombination-dependent replication restart, nor the recombination intermediates arising as a result of non-allelic HR activate the DNA damage or the intra-S phase checkpoint. Our analysis during a single defined synchronous cell cycle shows that HR-dependent replication intermediates arise in S phase, shortly after replication arrest. Thus, HR-dependent replication fork restart occurs in S phase. Interestingly, the recombination intermediates persisted until G2. This is consistent with the tight regulation of structure-specific nucleases, such as Mus81/Eme1: these are excluded from processing DNA structures in S phase, but become activated by CDK phosphorylation in G2 (refs 26, 27). The decline of the HR intermediates during G2 was coincident with an increase in capped chromosomes observed in late S and G2. This suggests that gross chromosomal rearrangements are generated in G2 through the resolution of the HR intermediates.

The capped chromosomes form bridges at anaphase and ‘cut’ cells arise during septation (cytokinesis), where the septum bisects the DNA. After breakage of the chromosome bridge during cytokinesis, the daughter cells each receive a chromosome fragment with an unrepairable one-ended DSB. While the DNA damage and the intra-S phase checkpoints are not activated in the first cell cycle after replication fork arrest, the breakage of the dicentric chromosome during cytokinesis leads to robust Chk1, but not Cds1^Chk2^ activation in the second cell cycle as cells proceeded into G2. This would be consistent with break resection exposing single-strand DNA and activating the DNA damage checkpoint. Break resection is known to be under control of CDK levels and becomes active in G2 (refs 30, 31, 32).

The segregation of one-ended DSBs into both daughter cells has analogies to the segregation of lesions resulting from replication stress into 53BP1 nuclear bodies in human G1 cells^33^,^34^. 53BP1 bodies are associated with regions such as CFS that have intrinsic replication difficulties^31^, but also originate from damage generated in mitosis. The 53BP1 nuclear bodies are usually symmetrically placed in pairs of newborn daughter cells^30^. In such cells, mitotic and/or cytokinesis-induced DSBs can be repaired in G1 by non-homologous end joining, an alternative route to the generation of aberrant chromosomes^35^. It has been proposed that 53BP1 nuclear bodies shield DNA ends until these can be resolved through recombination later in the cell cycle, for example in S phase^30^. In our fission yeast experiments we saw no delay to S phase (by either flow cytometry or 2D-gel analysis (Figs 1c and 3c)) in response to chromosome missegregation and breakage, which would be consistent with the expectation that 53BP1 protects ends for repair in S phase. However, since G1 in rapidly proliferating *S. pombe* cells is very short and septation is coincident with S phase, it is likely that chromosome breakage as a consequence of cytokinesis occurs early in S phase rather than in G1.

In conclusion, we demonstrate that recombination-dependent replication restart and recombination intermediates do not activate the DNA damage or the intra-S phase checkpoints in the first cell cycle to impose a mitotic delay. Thus, the DNA structure checkpoints are blind to events that cause GCRs and CIN, important drivers in tumorigenesis. The checkpoints cannot act to supress fragile site expression by delaying mitosis but function locally at the site of DNA metabolism^28^. This may help to explain why cells routinely enter mitosis with chromosome bridges and explain why specific pathways, such as the controlled processing and resolution of DNA structures arising in late replicating regions^36^, have evolved.

## Methods

### *S. pombe* strains

S. pombe strains used in the study are listed in Table 1.

### Synchronous cultures

Parallel ‘on’ and ‘off’ cultures were set up for each time course from the same preculture. Strains were grown in Edinburgh Minimal Media (EMM media) supplemented with adenine and leucine (EMM+AL) at 30 °C. A low log phase preculture was diluted and aliquoted into 5 × 500 ml uninduced cultures and grown to early log phase (1.25 × 10^6^ cells per ml). The cell cycle was blocked in mitosis by inactivation of cold sensitive *nda3-KM311* by shifting the temperature to 16 °C for 6 h. P_*urg1lox*_*-rtf1* expression was induced by the addition of uracil at 0.25 mg ml^−1^ to the ‘on’ culture (4/5 flasks) 1 h before release, the remaining flask left uninduced. The cells were released from the *nda3-KM311* block by shifting the temperature to 30 °C. *T*=0 was taken when the incubator reached 30 °C. For each time point after release samples were taken to monitor cell cycle progression by flow cytometry and septation index and for DNA extraction and/or protein extraction. Samples were taken from ‘arrest on’ flasks in rotation.

### Analysis of DNA intermediates

Chromosomal DNA was extracted using standard procedures embedded in agarose plugs and PFGE of whole chromosomes was carried out using the Biorad CHEF-DRIII system under the following conditions: Initial switch time 1800, s, final switch time 1800, s, angle 100°, 2 Vcm^−1^ for 48 h. In time courses for each time point 1.25 × 10^9^ cells were collected. DNA was digested in plugs using 30 units of restriction enzyme and analysed by 1D or 2D gels^37^. For 2D gel electrophoresis 0.35% and 0.9% agarose was used for the first and second dimensions, respectively. Southern blot hybridization was performed using standard procedures. Autoradiography was performed using a storage phosphor screen/Storm PhosphorImager system. Band intensities were quantified using ImageQuant and presented as a percentage of the total intensity of signals of each individual lane.

### Protein analysis

Cells (5 × 10^7^) were used for each time point and cell extracts were prepared by trichloroacetic acid (TCA) extraction^38^. Cells were pelleted, washed and resuspended in 200 μl of 20% TCA solution. Acid washed glass beads were added and cells lysed using a Ryboliser (FastPrep24, MP) at 6.5 m s^−1^ for 30 s (repeated 2–3 times). A volume of 400 μl of 5% TCA was added, and the cell homogenate was spun (4,000 r.p.m.) into a new test tube. Following centrifugation at 14,000 r.p.m. for 5 min, the supernatant was discarded and the pellet was resuspended in 200 μl of loading buffer (250 mM Tris-HCl (pH 8), 2% sodium dodecyl sulfate (SDS), 5% glycerol, 5% β-mercaptoethanol, 0.1% bromophenol blue). Boiled samples were analysed by SDS-polyacrylamide gel electrophoresis (PAGE) and western blotting. To detect the Cds1^Chk2^ phosphoshift a 7.5% resolving gel containing a final concentration of 20 μM Phostag (Alpha labs), and 40 μM MnCl_2_ was used and the gel run at 15 mA. Mouse monoclonal anti-HA (Santa Cruz Biotechnology) diluted 1:5,000 was used to detect Chk1-HA^39^ and rabbit polyclonal anti-Cds1^40^ diluted 1:1,000 to detect Cds1. As a loading control, rabbit polyclonal anti-Cdc2 antibody (diluted 1:5,000; Santa Cruz) was used. Uncropped scans of western blots in Fig. 4 are shown in Supplementary Fig. 7.

## Author contributions

S.M., K.M. and A.W. performed the experiments. J.M.M. and A.M.C. wrote the manuscript. All authors contributed to the experimental design.

## Additional information

**How to cite this article:** Mohebi, S. *et al.* Checkpoints are blind to replication restart and recombination intermediates that result in gross chromosomal rearrangements. *Nat. Commun.* 6:6357 doi: 10.1038/ncomms7357 (2015).

## Supplementary Material

Supplementary Information Supplementary Figures 1-7 and Supplementary References

## Figures and Tables

**Table 1:** *S. pombe* strains used in the study.

**Strain**	**Genotype**	**Reference**
YSM072	*h+ ura4-D18 leu-32 urg1_NR::HPH rtf1::natMX6 nda3-KM311*	This study
YSM091	*h- smt0 RuiuR leu-32 urg1_NR::rtf1:DSR rtf1::natMX6*	This study
YSM092	*h- smt0 RuiuR leu-32 urg1_NR::rtf1:DSR rtf1::natMX6 nda3-KM311*	This study
YSM093	*h- smt0 RuiuR leu-32 urg1_NR::rtf1:DSR rtf1::natMX6 nda3-KM311*	This study
YSM094	*h- smt0 RuiuR leu-32 urg1_NR::rtf1:DSR rtf1::natMX6 nda3-KM311*	This study
YSM095	*h- smt0 RuiuR leu-32 urg1_NR::rtf1:DSR rtf1::natMX6 nda3-KM311*	This study
YSM096	*h- smt0 RuiuR leu-32 urg1_NR::rtf1:DSR rtf1::natMX6 nda3-KM311*	This study
YSM097	*h- smt0 RuiuR leu-32 urg1_NR::rtf1:DSR rtf1::natMX6 nda3-KM311*	This study
YSM098	*h- smt0 RuiuR leu-32 urg1_NR::rtf1:DSR rtf1::natMX6 nda3-KM311*	This study and ref. 22
YSM101	*h- smt0 RuiuR leu-32 ade6-704 urg1_NR::HPH rtf1::natMX6 nda3-KM311 Chk1-3HA*	This study
YSM130	*h- smt0 RuiuR leu-32 ade6-704 urg1_ NR::rtf1:DSR rtf1::natMX6 nda3-KM311 Chk1-3HA*	This study
YKM041	*h- smt0 RuiuR leu1-32 ade6-704 sup3.5:nmt41:rtf1*	ref. 20
YKM125	*h- smt0 RuiuR leu1-32 ade6-704 sup3.5:nmt41:rtf1 rad3::kanMX6*	This study
YKM145	*h- smt0 RuiuR leu1-32 ade6-704 sup3.5:nmt41:rtf1 rad3::hphMX6*	This study
YKM151	*h- smt0 RuiuR leu1-32 ade6-704 sup3.5:nmt41:rtf1 cds1::natMX6*	This study
YKM153	*h- smt0 RuiuR leu1-32 ade6-704 sup3.5:nmt41:rtf1 ckh1::kanMX6*	This study
YKM791	*h-smt0 nda3-KM311 ade6-704 ura4-d18 leu1-32*	This study
