# Supplementary material for: Checkpoints are blind to replication restart and recombination intermediates that result in gross chromosomal rearrangements
Source: Nat Commun. 2015 Feb 27;6:6357. doi: 10.1038/ncomms7357 (PMC4351560; doi:10.1038/ncomms7357)
Supplement: Supplementary Information — Supplementary Figures 1-7 and Supplementary References [file ncomms7357-s1.pdf]

Supplementary Figure 1

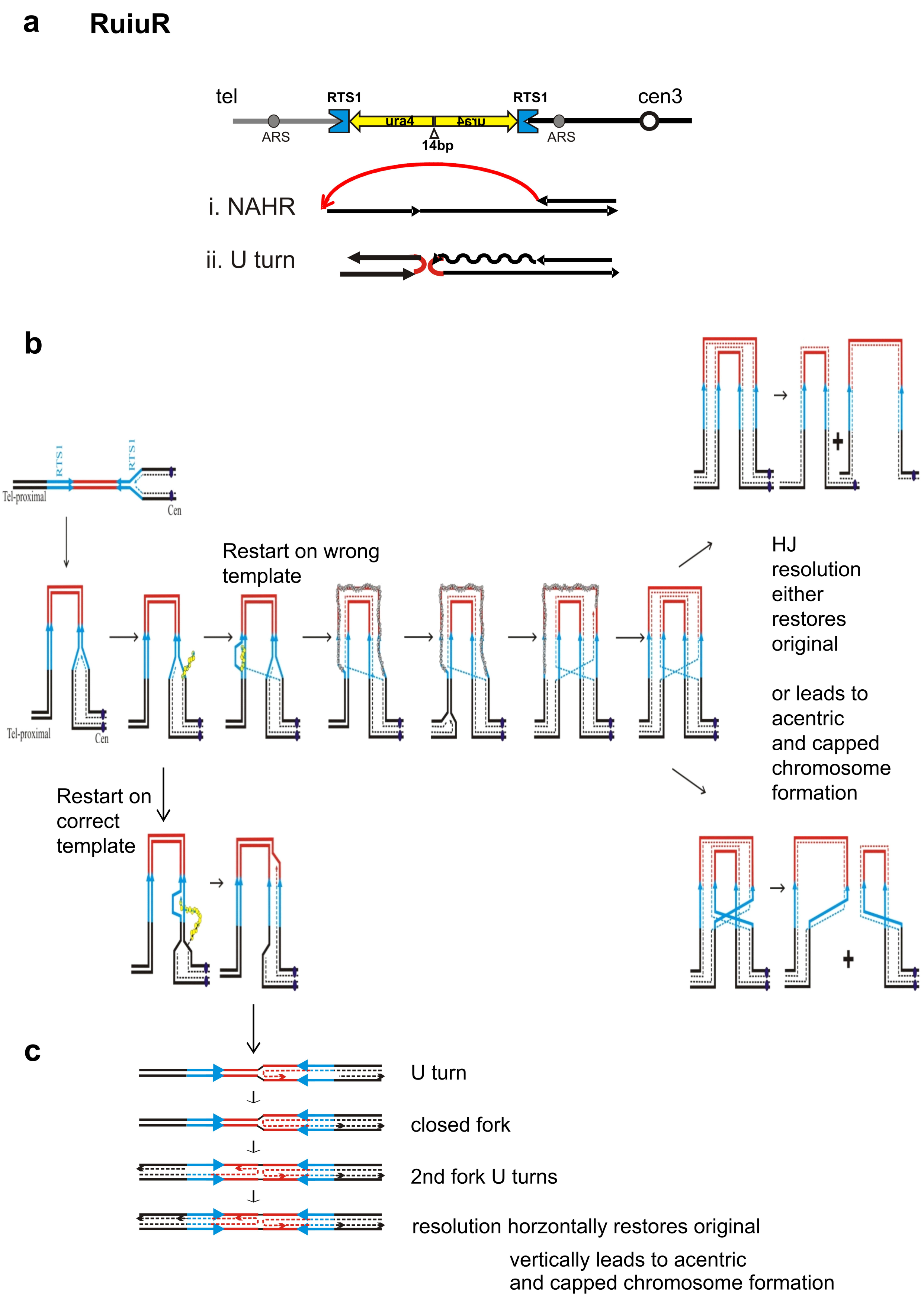

Supplementary Figure 1. Mechanisms for HR-dependent replication restart generating genomic rearrangements.

(**a**) Cartoon of palindrome replication stall system (RuiuR). Cen3 denotes centromere 3. Concave blue boxes represent *RTS1*, replication termination sequences as indicated. Yellow arrows represent *ura4* sequences and orientation. Open triangle shows 14-bp interrupting sequence at the palindrome centre. HR-dependent replication restart generates palindromic acentric and capped chromosomes by two mechanisms: **i**) Non-allelic homologous recombination (NAHR), where HR-dependent restart occurs on the wrong template[1, 2] and **ii**) HR-dependent restart on the correct template but the restarted fork then U-turns at the centre of the palindrome[3]. (**b**) Use of an ectopic sequence to template HR-dependent replication restart leads to rearrangements generating acentric and capped chromosomes. Our model suggests that the nascent strand becomes single stranded behind a collapsed fork, associates with HR proteins and subsequently anneals with the correct template to resume replication (panel below). However, if a DNA sequence homologous to the collapse site is nearby, an erroneous invasion of the nascent strand can occur, causing template exchange such that replication reinitiates ectopically[1, 2]. In these situations the restarted forks exchanged templates resulting in single (shown) or double Holliday Junctions (HJs) (not shown) between the *RTS1* sequences. An intact single HJ is a four-way junction and coordinated symmetrical cleavage would lead to unbiased resolution in one of two planes: resolution in one plane results in two identical sister chromatids, but resolution in the second plane generates inverted chromosomal fusions manifesting as acentric and capped chromosomes. Double HJs resolution can additionally lead to inversion of the intervening *ura4* sequence[2]. (**c**) HR-dependent restart on the correct template also leads to rearrangements generating acentric and capped chromosomes. Our data[3] showed that the HR-restarted fork is non-canonical and error prone causing GCRs at inverted repeats due to executing a U-turn. The incoming fork from the other direction is then forced to U-turn. This generates a Holliday junction-like structure that can be resolved in one of two planes either regenerating the original chromosome conformation or generating capped and acentric chromosomes.

Supplementary Figure 2

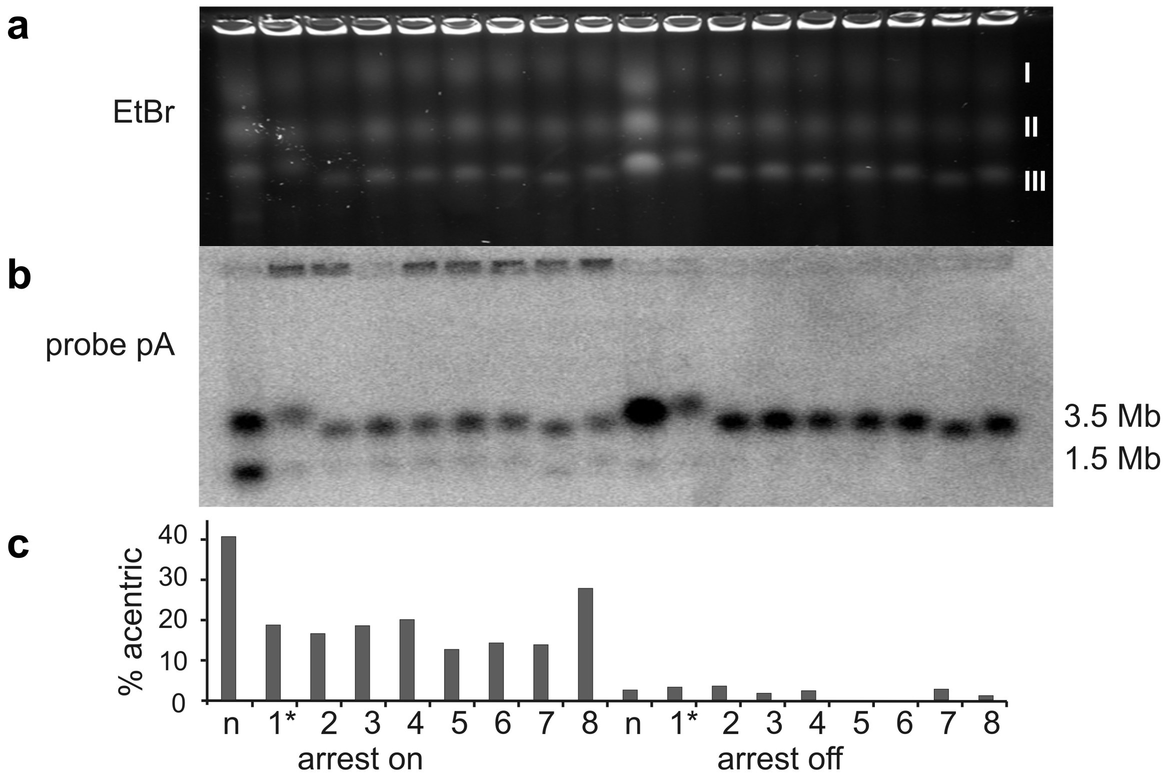

Supplementary Figure 2. HR-dependent replication restart leads to high levels of GCR

Pulse field gel electrophoresis of the three *S. pombe* chromosomes shows that, following induction of fork arrest, acentric chromosomes increase by 15.8% (arrest "on" mean 17.95%±4.86; arrest "off" mean 2.15%±1.35) 3 hrs after the induction of replication arrest. (**a**) Ethidium bromide stained gel. (**b**) Probe pA (telomeric to RuiuR) hybridises to the 3.5 Mb chromosome III and to the 1.5 Mb acentric chromosome. Note that chromosome III has rDNA repeats at both ends and the size of the chromosome and acentric varies with rDNA repeat number. (**c**) Quantification of acentric signal relative to total pA signal. The ratio of the acentric palindromic chromosome to total ChrIII signal is indicated as a percentage and was calculated as 100 × 1.4 Mb/(2 × 3.5 Mb + 1.4 Mb) or 100 × 10.3 kb/(2 × 19.8 kb + 10.3 kb). Note the duplication of the probe sequence in the acentric. n designates the original *nmt41-rtf1* RuiuR system[1], 1-8 are independent isolates of *urg1-rtf1* RuiuR, 1* in an *nda3+* background and 2-8 in the *nda3-KM311* background. Note that *nda3-KM311* does not affect the levels of chromosome rearrangement. 'Arrest on' for *nmt-rtf1* is 24 hrs after removal of thiamine as the *nmt* promoter takes approximately 16 hrs to induce and thus represents the steady state levels of acentric chromosomes over several cell cycles. 'Arrest on' for the *urg1-rtf1* isolates is 3 hrs after the addition of uracil, which, since the majority (~70%) of cells are in G2 and the cell cycle duration in minimal media at 30°C is approximately 3 hrs, is equivalent to passage through a single S phase.

Supplementary Figure 3

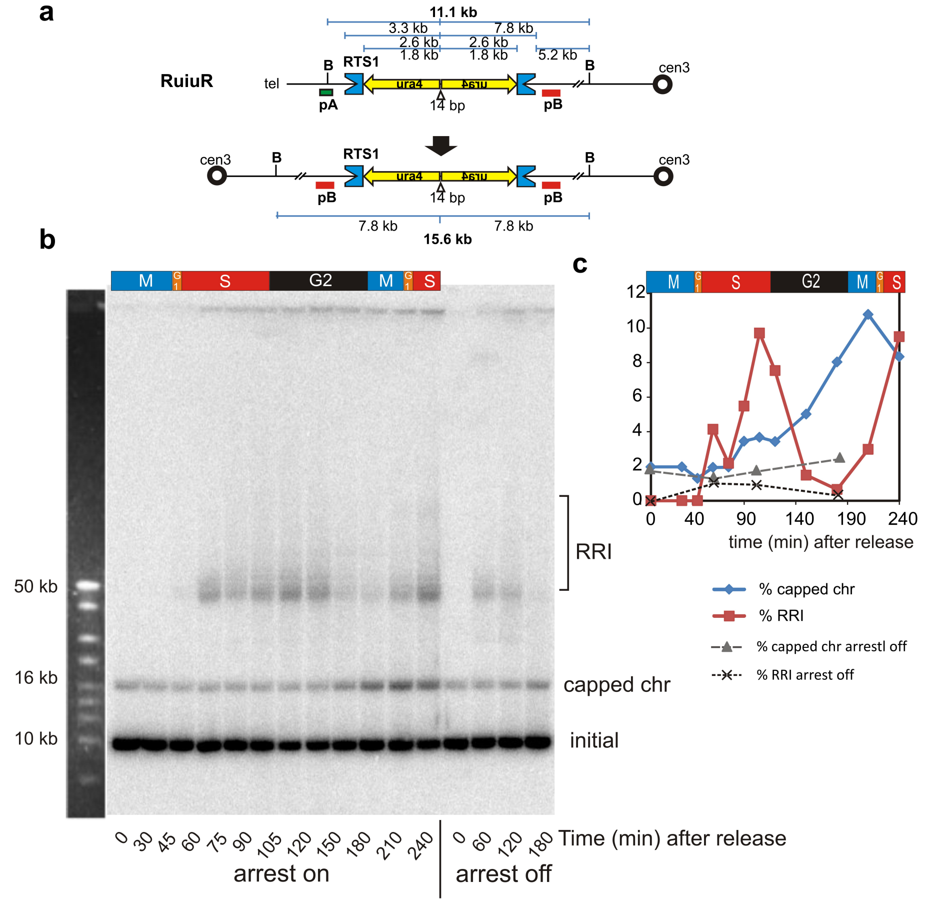

Supplementary Figure 3. Analysis of genomic rearrangements through the cell cycle

(**a**) Cartoon of palindrome replication stall system (RuiuR). Cen3 denotes centromere 3. Concave blue boxes represent *RTS1*, replication termination sequences as indicated. Yellow arrows represent *ura4* sequences and orientation. Open triangle shows 14-bp interrupting sequence at the palindrome centre. Red bars represent probes. B indicates *Bgl*II restriction site. Sizes of initial and predicted dicentric chromosome *Bgl*II fragments are shown. Green bar pA and red bar pB denote the positions of telomeric and centromeric probes respectively. (**b**) Southern blot analysis of an independent time course to that shown in Fig. 2 with samples taken at designated minutes after release from mitotic block with Rtf1 induced (arrest on) or uninduced (arrest off). Genomic DNA was digested with *Bgl*II and probed with pB. (**c**) Quantification of rearranged fragment in **b**. The 7.4 kb capped chromosome fragment increases from background levels after 150 min. Slow migrating replication stall and recombination intermediates (RRI) at seen from 60-120 min in the 'on' culture, coincident with S phase, reducing as the level of the capped chromosome increases in G2 and returning at 210-240 min, coincident with the second S phase. In the parallel uninduced (arrest off) cultures the capped chromosome levels remain constant.

Supplementary Figure 4

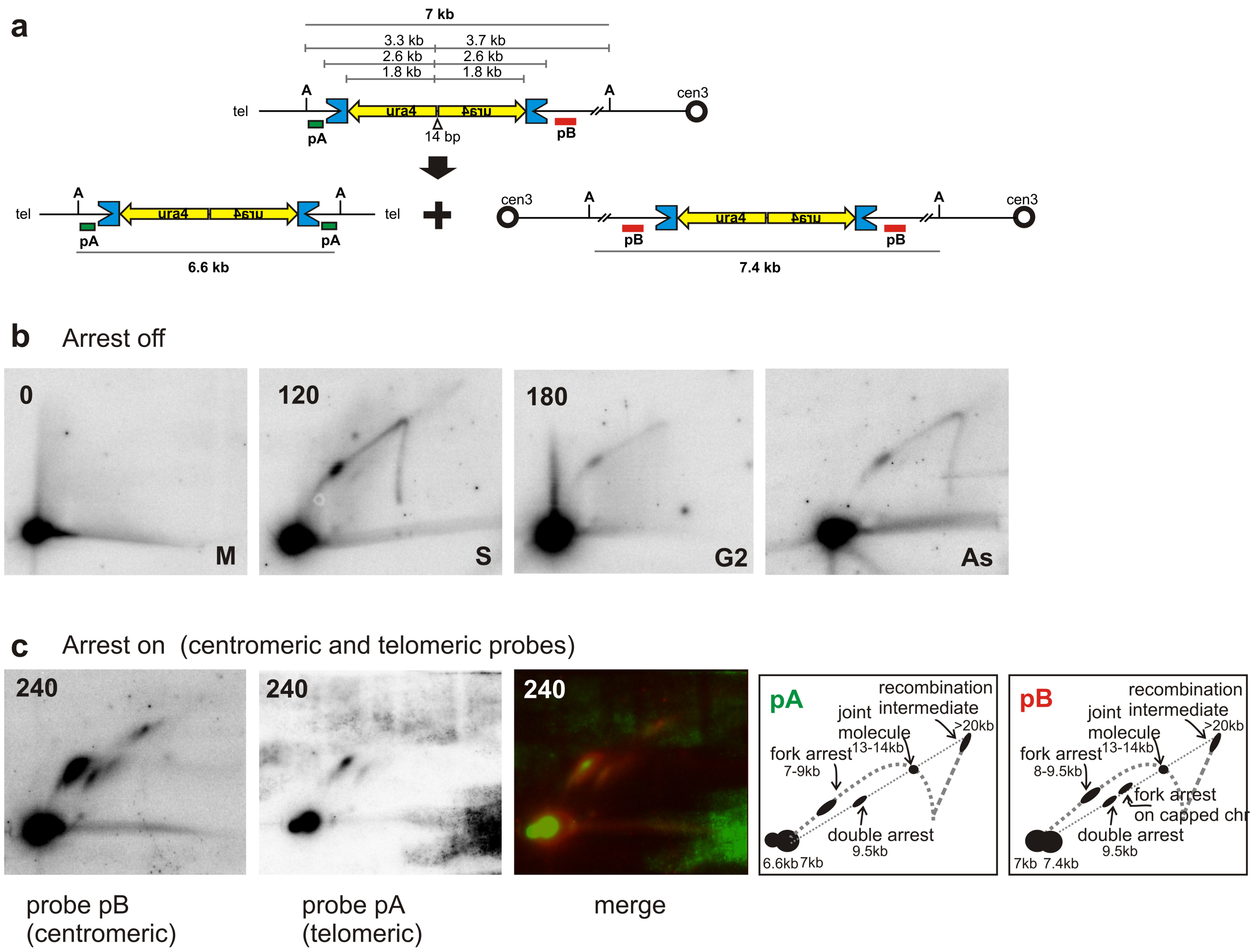

Supplementary Figure 4. The capped chromosome replicates in the second cell cycle

(**a**) Cartoon of RuiuR replication stall system. Cen3 denotes centromere 3. Concave blue boxes represent *RTS1*, replication termination sequences. Yellow arrows represent *ura4* sequences and orientation. Open triangle shows 14-bp interrupting sequence at the palindrome centre. Red bars represent probe pB, green bar probe pA. A indicates *Ase*I restriction site. Sizes of initial and predicted acentric and capped chromosome *Ase*I fragments are shown. (**b**) Two-dimensional gel electrophoresis of replication intermediates from samples taken at designated minutes after release from mitotic block in an uninduced (arrest off) culture. Genomic DNA was digested with *Ase*I and probed with pB (centromeric to palindrome). A monomer spot (bottom right), corresponding to unreplicated DNA is seen in the mitotic sample (T=0). The Y-arc, indicative of passive replication through the region, is seen in the S phase sample (120 min) and a faint pause spot, consistent with leakiness of the *urg1* promoter in the off state. These intermediates are much reduced by 180 min when cells are in G2. No new intermediates are seen in the asynchronous (As) sample. (**c**) Two-dimensional gel electrophoresis of replication intermediates from induced sample 240 min after release from mitotic block and cartoons of replication intermediates visualised with probes pA (telomeric) and pB

(centromeric). Genomic DNA was digested with *Ase*I and probed sequentially with probe pB and probe pA. In the merged picture the signal corresponding to the telomeric probe is green, the centromeric probe is red (yellow when both probes hybridise). Red and green spots flanking the yellow monomer spot correspond to capped chromosome and acentric rearrangements. The spot to the right of the double pause site is only detected by the centromeric probe pB, and is of a size that correlates with replication pausing on the capped chromosome in the second cell cycle. We note that this analysis does not distinguish between replication on a chromosome fragment resulting from breakage of the anaphase bridge or an intact capped chromosome.

Supplementary Figure 5

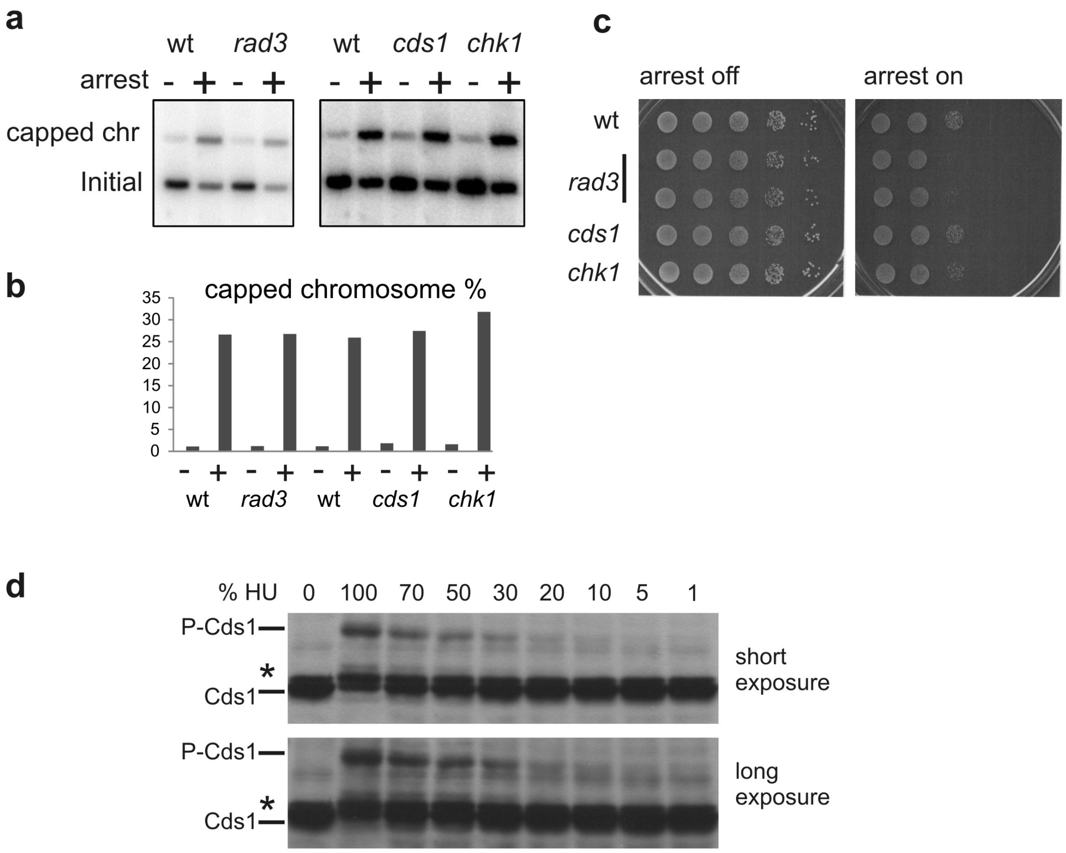

Supplementary Figure 5. DNA integrity checkpoints are not required for HR-dependent replication restart and the generation of chromosome rearrangements.

(**a**) Southern blot analysis of chromosome rearrangements in checkpoint mutants after induction of replication arrest in the palindrome system. Rtf1 was expressed under the control of the *nmt41* promoter for 24hrs in media lacking thiamine (+) and compared to uninduced (-) cells grown in the presence of thiamine in wild type (wt), *rad3*, *cds1* or *chk1* null cells. Genomic DNA digested was with *Bgl*II. (**b**) Quantification of the 15.6kb capped chromosome band in shows that chromosome rearrangements are unaffected in checkpoint mutants. (**c**) Cell survival after induction of replication arrest in the palindrome system in wild type (wt), *rad3*, *cds1* or *chk1* null cells. Rtf1 was expressed under the control of the *nmt41* promoter for 24hrs in media lacking thiamine and compared to uninduced cells grown in the presence of thiamine. Serial dilutions of cells were plated and incubated for 3 days at 30°C. Induction of arrest causes loss of viability in all strains but this was slightly increased in *rad3-d*. (**d**) Quantification of sensitivity of the Cds1 phosphorylation assay. Extracts from HU arrested were serially diluted with an extract from untreated asynchronous cells. The phosphorylated band was detectable when 1% of the extract was from cells arrested in HU.

Supplementary Figure 6

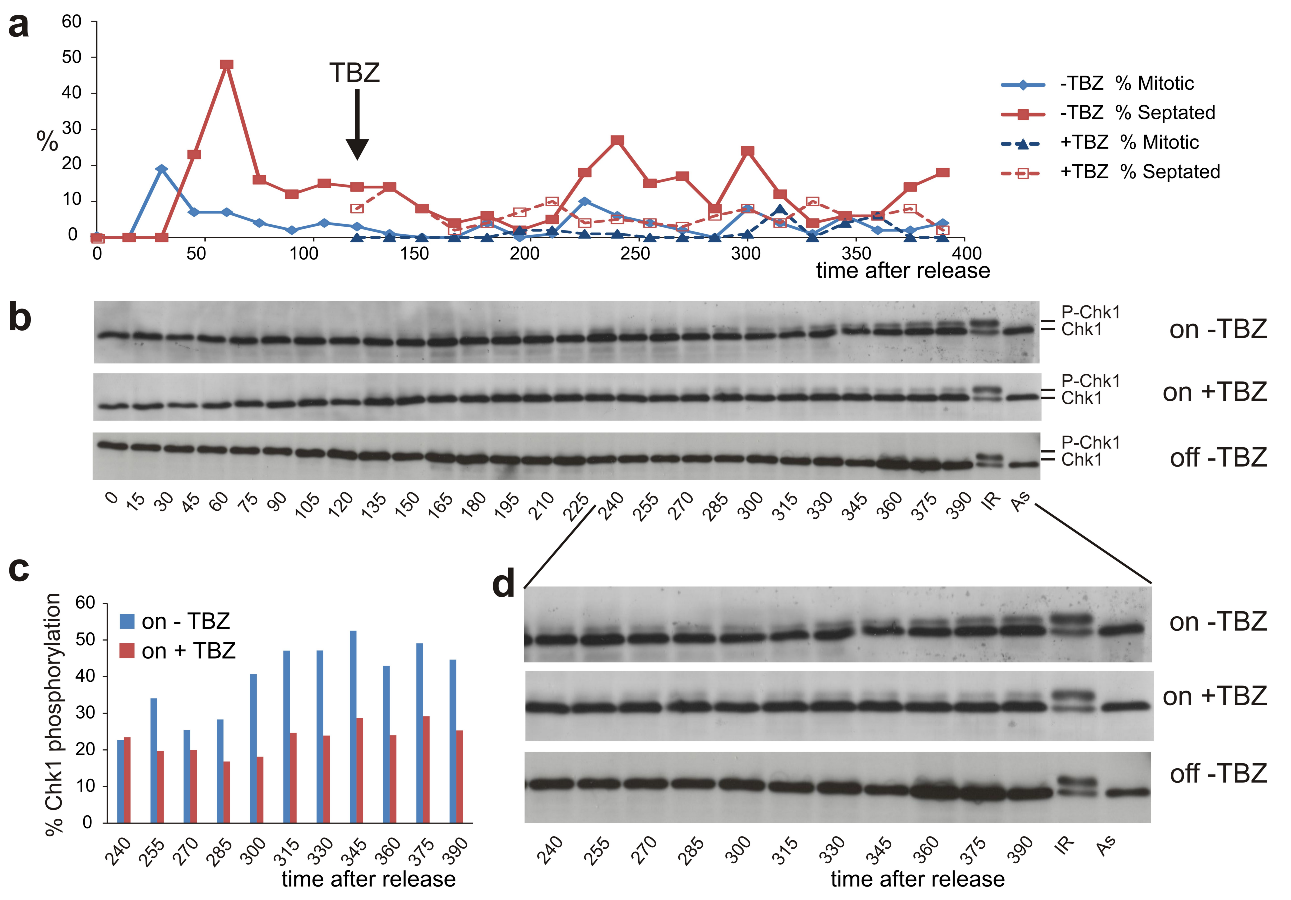

Supplementary Figure 6. DNA damage checkpoint activation in the second cell cycle is dependent on passage through mitosis.

(a) Profile of cell cycle progression shown for a representative experiment (n=3) by percentage of mitotic (M) and septated (S) cells after Rtf1 induction and release from mitotic block. Thiabendazole (TBZ), which is a mitotic spindle inhibitor, was added to half the culture at 120 min when the cells were in G2. (**b**) Western blot analysis of Chk1 (DNA damage effector kinase) activation at designated times through the cell cycle following release from mitotic block in induced cultures with and without TBZ (on +TBZ and on -TBZ, respectively) and a parallel uninduced (off -TBZ) culture. (**c**) Quantification of Chk1 phosphorylation. (**d**) Enlargement of later time points from (**b**). IR indicates cells treated with 200 Gy and As asynchronous controls. HA-Chk1 was detected by anti-HA antibodies. No Chk1 phosphorylation was seen in the first cell cycle following release. In the second cell cycle phosphorylated Chk1 was detected in the untreated induced culture but this was reduced in the presence of TBZ and absent in the uninduced culture. Thus, checkpoint activation is dependent on passage through mitosis after replication stalling is induced, consistent with damage generated in mitosis activating the checkpoint in the second cell cycle.

Supplementary Figure 7

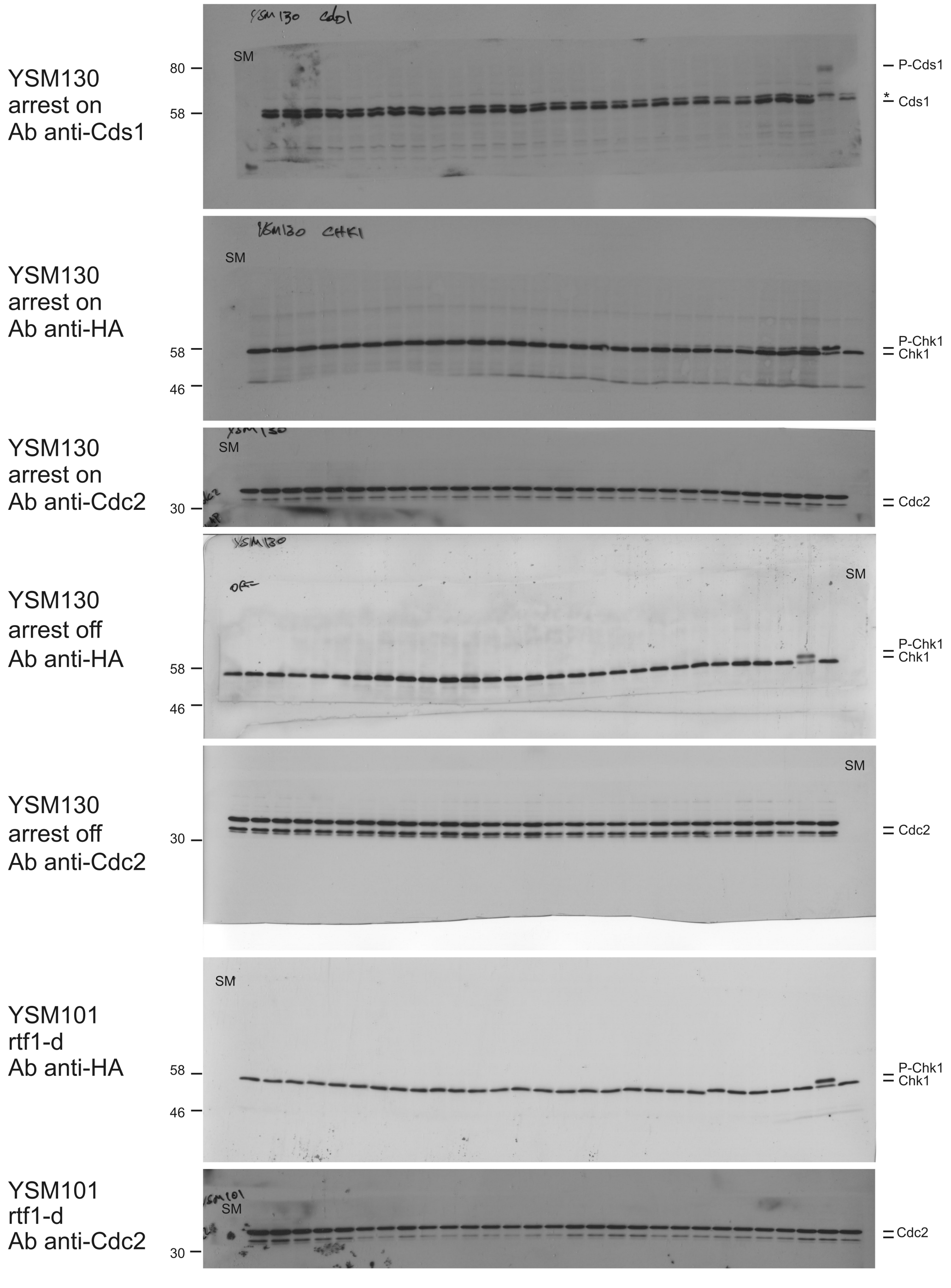

Supplementary Figure 7. Uncropped westerns for Fig.4

Profiles of Cds1^{Chk2} (S phase effector kinase) and Chk1 (DNA damage effector kinase) activation through the cell cycle following release from mitotic block in induced (YSM130 arrest on), uninduced (YSM130 arrest off) and no arrest (YSM101 rtf1-d) cultures. * indicates a non-specific band. Cds1 was detected using anti-Cds1 antibodies, Chk1-HA by anti-HA, Cdc2 loading control by anti-Cdc2 antibodies. SM indicates the position of the lane containing the size marker. The relevant positions for the closest markers are indicated for each blot on the left. The prestained broad range marker (New England Biolabs) did not cross react with the antibodies used.

Supplementary References

1. Mizuno, K., Lambert, S., Baldacci, G., Murray, J.M. & Carr, A.M. Nearby inverted repeats fuse to generate acentric and dicentric palindromic chromosomes by a replication template exchange mechanism. *Genes Dev* **23**, 2876-2886 (2009).
2. Lambert, S. *et al.* Homologous recombination restarts blocked replication forks at the expense of genome rearrangements by template exchange. *Mol Cell* **39**, 346-359 (2010).
3. Mizuno, K., Miyabe, I., Schalbetter, S.A., Carr, A.M. & Murray, J.M. Recombination-restarted replication makes inverted chromosome fusions at inverted repeats. *Nature* **493**, 246-249 (2013).
